# Bat Flies and Their Microparasites: Current Knowledge and Distribution

**DOI:** 10.3389/fvets.2019.00115

**Published:** 2019-04-24

**Authors:** Tamara Szentiványi, Philippe Christe, Olivier Glaizot

**Affiliations:** ^1^Department of Ecology and Evolution, University of Lausanne, Lausanne, Switzerland; ^2^Museum of Zoology, Lausanne, Switzerland

**Keywords:** bat flies, microparasite, chiroptera, pathogen, distribution

## Abstract

Bats are the second most diverse mammalian group, playing keystone roles in ecosystems but also act as reservoir hosts for numerous pathogens. Due to their colonial habits which implies close contacts between individuals, bats are often parasitized by multiple species of micro- and macroparasites. The particular ecology, behavior, and environment of bat species may shape patterns of intra- and interspecific pathogen transmission, as well as the presence of specific vectorial organisms. This review synthetizes information on a multi-level parasitic system: bats, bat flies and their microparasites. Bat flies (Diptera: Nycteribiidae and Streblidae) are obligate, hematophagous ectoparasites of bats consisting of ~500 described species. Diverse parasitic organisms have been detected in bat flies including bacteria, blood parasites, fungi, and viruses, which suggest their vectorial potential. We discuss the ecological epidemiology of microparasites, their potential physiological effects on both bats and bat flies, and potential research perspectives in the domain of bat pathogens. For simplicity, we use the term microparasite throughout this review, yet it remains unclear whether some bacteria are parasites or symbionts of their bat fly hosts.

## Introduction

Bats are the second most diverse mammalian group after rodents, with ~1,390 recognized species across 227 genera (1). Many bat species play keystone roles in ecosystems, where they are essential to pollination, seed dispersal, and pest control (2). Several studies have also highlighted their prominent role as pathogen-reservoirs (3, 4); viruses being the best studied due to their potential as human pathogens (3, 5–8). Bats host more viruses per species than rodents, making them an interesting system for both disease ecology and public health research (4, 9).

Bacteria (such as *Bartonella* spp. and *Borrelia* spp.) and protozoans (such as *Trypanosoma* spp. and *Plasmodium* spp.) have also been detected in bats (8, 10, 11). In recent years, bat-associated *Bartonella* genotypes have been found in humans, indicating the public health importance of this parasite in bats (12–14). *Bartonella* and other pathogen transmission from bats to humans may occur through religious activities in caves, bat consumption or contact with contaminated products (12, 15). There are documented cases of bat-specific ectoparasites biting humans (16, 17), increasing the potential of bat-born pathogen transmission. Additionally, bat-associated pathogen, such as *Trypanosoma cruzi* genotype has also been found in humans (18).

Bats host numerous ectoparasitic groups, such as bat flies (Diptera: Nycteribiidae and Streblidae), bugs (Hemiptera: Cimicidae and Polyctenidae), fleas (Siphonaptera: Ischnopsyllidae), and several bat specialized arachnids, such as mites (Mesostigmata: Spinturnicidae and Macronyssidae) and ticks (e.g., *Argas* spp., *Carios* spp., *Ixodes* spp., and *Ornithodoros* spp.) (19–25).

**Figure 1:**
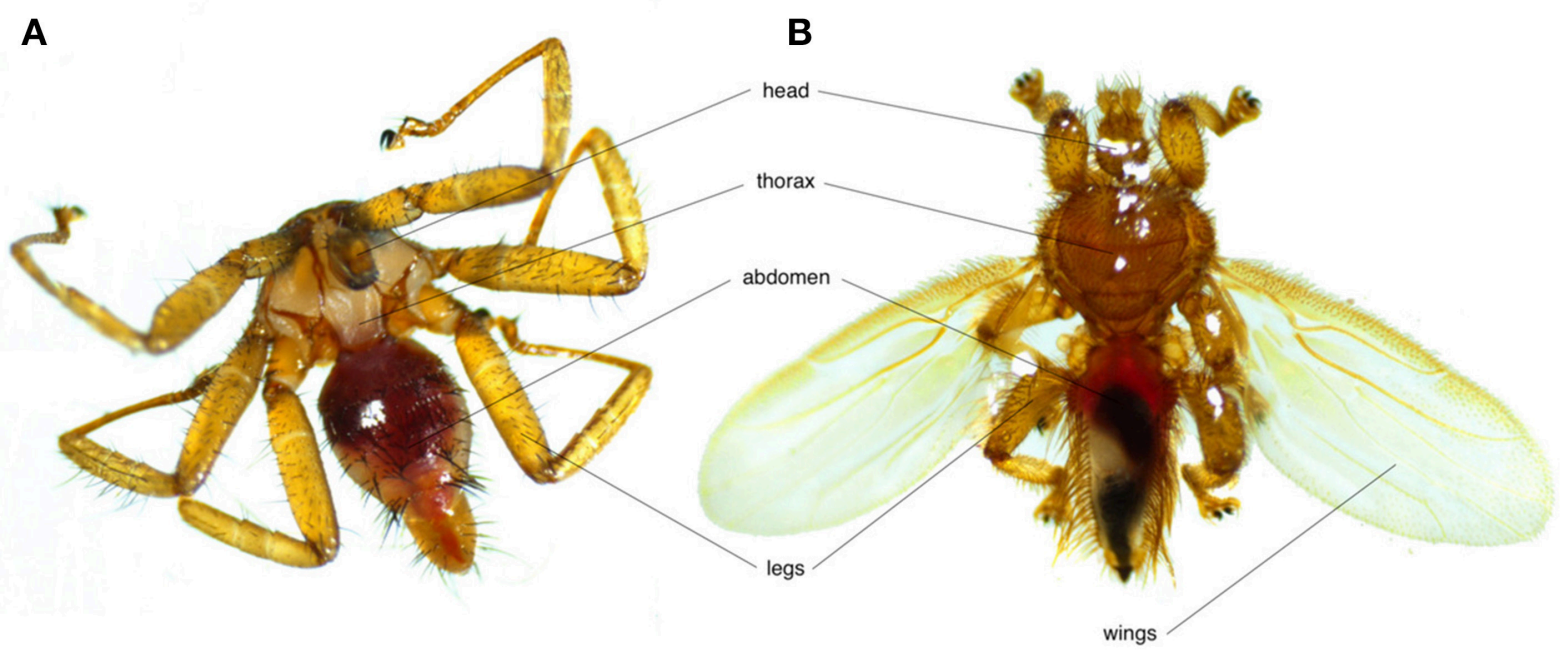
Photos showing the morphological differences between **(A)** a wingless nycteribiid and **(B)** a streblid bat fly.

Bat flies (Nycteribiidae and Streblidae) are the most common bat ectoparasites (Figure 1). Both families, along with Hippoboscidae (louse and ked flies) and Glossinidae (tsetse flies) belong to the Hippoboscoidea superfamily. Currently 275 species across 21 genera of nycteribiids and 227 species across 31 genera of streblids are recognized. Nycteribiids have a higher diversity in the Eastern Hemisphere, while streblids are mainly found in the Western Hemisphere (17).

Members of Hippoboscoidea have developed a unique reproductive strategy. A single larva develops within a female, feeding on the secretion of the so-called milk glands. Larviposition occurs at the third instar stage and the larva immediately pupates. The four families have thus been previously referred as “Pupipara” (an obsolete clade). This unique reproductive strategy necessitates milk gland secretion transfer for larval development (26–28), which may shape the community of certain bacteria such as *Arsenophonus, Bartonella*, or *Wolbachia* by vertical transmission (26, 27, 29, 30). Horizontal transmission may occur through parasitoids or individuals contacting contaminated saliva, as in plant consuming insect communities (31, 32).

Bat flies deposit their larva on substrates such as the host roost wall. After larviposition, females return to their host. When the offspring emerge, they actively search for bat hosts. Emergence time depends on several factors including temperature and host presence (33, 34). Regarding their reproductive strategy, bat flies also show strong morphological adaptations to their parasitic life style. Some species are eyeless or have reduced facets (35). Nycteribiids are wingless, while most streblid species have partly or fully developed wings.

Early studies assumed that bat flies show no strong host specificity (36, 37); nevertheless more comprehensive recent works showed that the majority of bat fly species exhibit high specificity to a single or closely related bat species when collection is controlled and contamination avoided (25, 38–41).

Bats' ectoparasites may have vectorial potential. For example, *Polychromophilus* spp. are transmitted by nycteribiids (42) and *Trypanosoma* spp. by cimicids (43). Although, the transmission route of *Bartonella* has not been experimentally tested, this bacteria has been detected in a wide range of bat ectoparasites, such as bat flies (44–46), tick, and mites (47–51). In a recent study, ectoparasite burden was shown to positively correlate with *Bartonella* infection, suggesting their potential role as vectors (52). Furthermore, *Bartonella* was detected in bat flies and their host in the Madagascan fruit bat (*Eidolon dupreanum*), but not in fleas, indicating the potentially crucial role of bat flies in *Bartonella* transmission (53). Additionally, ectoparasite and virus species richness positively correlate, suggesting a vectorial role of ectoparasites for viruses (54).

In this review we focus on bat flies, the most diverse and prevalent group of bat ectoparasites. Bat flies are common on most species and since they are obligate hematophagous dipterans, they may play an important role in the transmission and maintenance of bat pathogens. The exact nature of the interaction between some bacteria and their bat fly hosts is unknown: *Wolbachia* and *Arsenophonus* may act as parasites and/or as mutualists (55, 56) (we consider them as potential microparasites in this review).

Here we review the presence of microparasites in bat flies and their geographical distribution. We consider the following organisms as microparasites: blood parasites, represented by *Polychromophilus* spp. and the extinct genus *Vetufebrus* sp. (Haemosporidia: Plasmodiidae); bacteria, such as *Arsenophonus* and *Providencia* (Enterobacteriales: Enterobacteriaceae), *Bartonella* (Rhizobiales: Bartonellaceae), *Wolbachia* and *Rickettsia* (Rickettsiales: Anaplasmataceae and Rickettsiaceae); viruses, such as Kanyawara virus (Mononegavirales: Rhabdoviridae), Mahlapitsi virus (Reoviridae), Wolkberg virus and Kaeng Khoi virus (Bunyavirales: Bunyaviridae and Peribunyaviridae), dengue virus (Flaviviridae); hyperparasites, such as fungi (Ascomycota: Laboulbeniaceae) and finally parasitoids (Hymenoptera: Eupelmidae). We test whether bat host phylogenetic origin effects the presence of different microparasitic groups of bat flies. We discuss the potential physiological effects of microparasites on both bats and bat flies, and future research perspectives related to bat-associated ectoparasites and microparasites.

## Materials and Methods

We present microparasite data collected from various literature source (Supplementary Data Sheet 1). We searched Google Scholar and ISI Web of Science, using all combinations of the following terms in English and French: Chiroptera or bat^*^; ectoparasite, bat fly, Nycteribiidae, Streblidae or Hippoboscidae^*^; and pathogen, parasitoid, parasite, microparasite, fungi, protozoa, haemosporidians, bacteria or virus.

Each bat fly—microparasite association (genus or species, depending on the taxonomic level provided by the authors) is an entry of the dataset, and is characterized by its geographical origin and bat host species.

We use currently valid taxonomical names for both bats and bat flies in our database (57–59). Statistics are conducted using R 3.5.1 (60). Bat fly-microparasite networks were visualized using the R package bipartite (61). Map of reported bat fly-microparasite associations were made in QGIS 2.16 (62).

## Results

### Effect of Bat Host Family on Detected Microparasite Distribution in Bat Flies

Bat flies infected with microparasites were observed on 75 bat species comprising 33 bat genera, with most in Vespertilionidae (16/505 known species), Phyllostomidae (21/216), Pteropodidae (13/196), Miniopteridae (10/38, the highest observed ratio), and Rhinolophidae (8/103). Bat flies with microparasite observations were also found in only a few species of Emballonuridae, Hipposideridae, Noctilionidae, and Mormoopidae.

**Figure 2:**
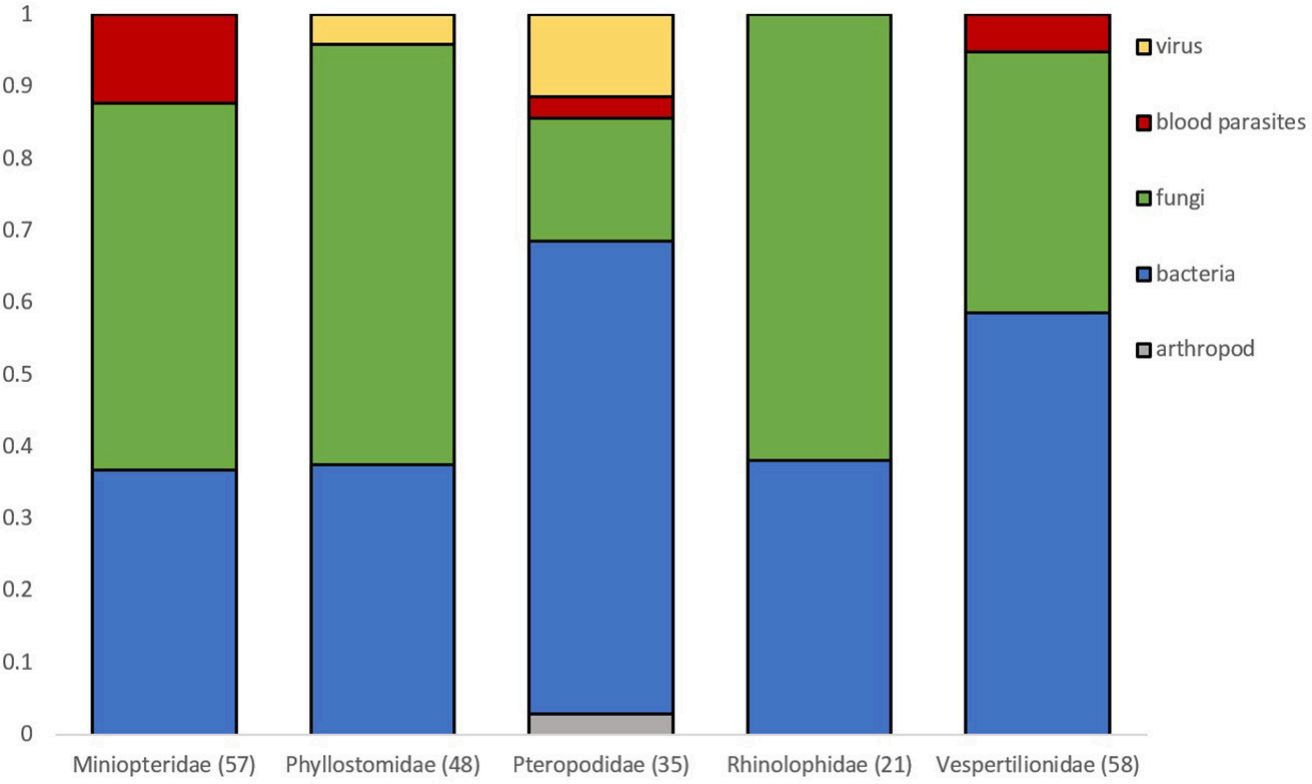
Proportion of microparasite groups observed in bat flies collected from different bat host families. Numbers in brackets are sample sizes. Families with < 20 observations are not represented.

Microparasite distribution in bat flies is dominated by bacterial and fungal parasites (Figure 2). Viruses detected in bat flies are only known from the family Phyllostomidae (*n* = 2) and Pteropodidae (*n* = 4). Blood parasites were mostly in flies from Miniopteridae (*n* = 7), but were also found in Pteropodidae (*n* = 1) and Vespertilionidae (*n* = 2) (Figure 2).

### Diversity Within Nycteribiidae and Streblidae

**Figure 3:**
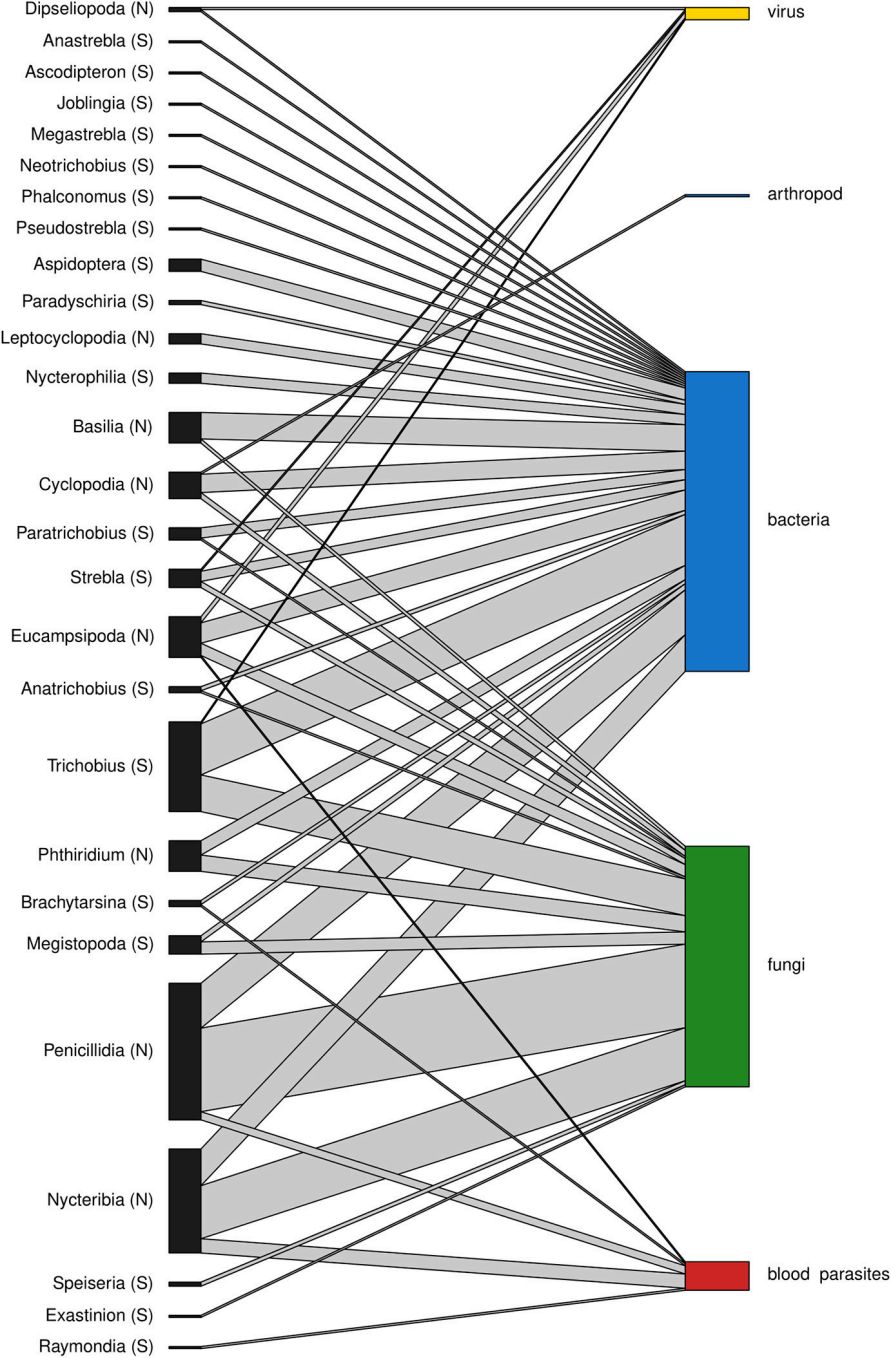
Association between bat fly genera of Nycteribiidae (N) and Streblidae (S) families and microparasitic groups. The height of the bars represents the relative abundance of the groups within each network level.

**Figure 4:**
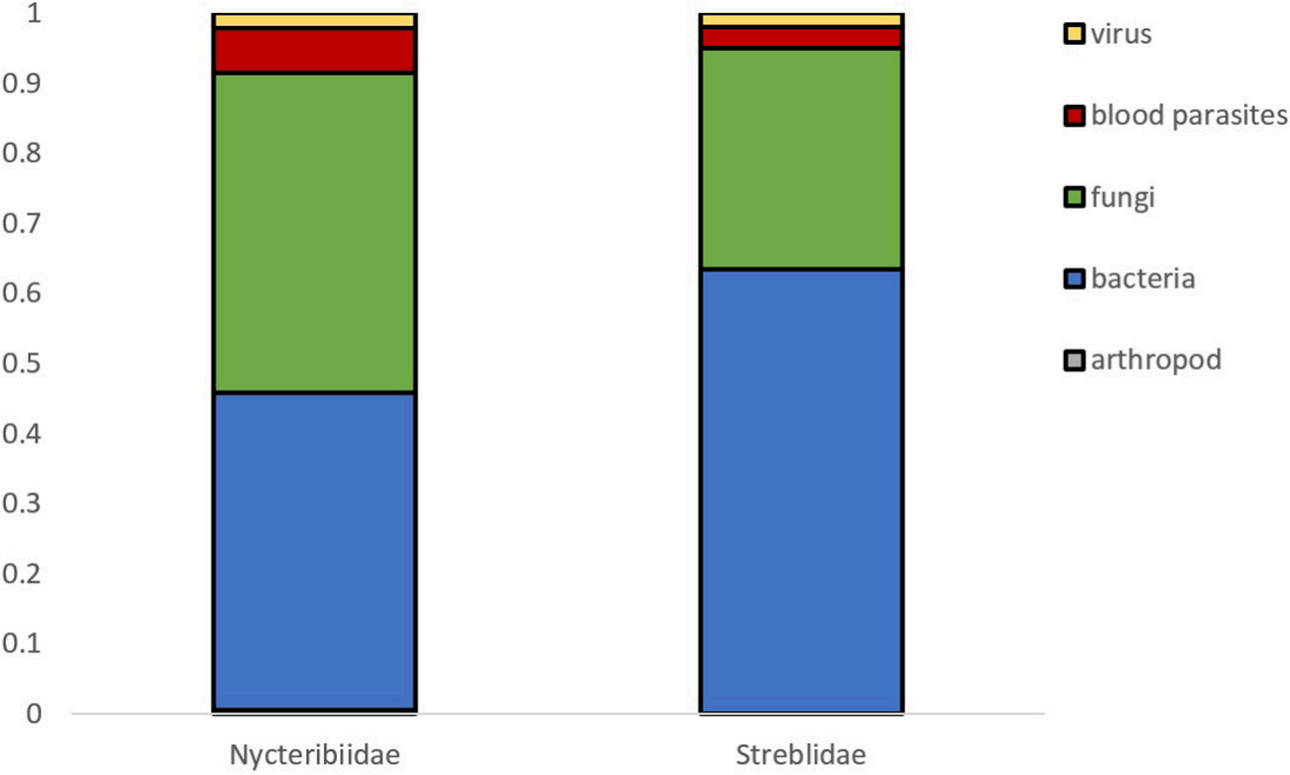
Microparasite distribution in both bat fly families.

A total of 188 and 101 microparasite observations are reported in bat fly families Nycteribiidae and Streblidae respectively, belonging to 27 bat fly genera (Figure 3). The most frequently reported infected bat fly genera are *Penicillidia* (*n* = 67), *Nycteribia* (*n* = 51), *Trichobius* (*n* = 44), *Eucampsipoda* (*n* = 20), and *Basilia* (*n* = 15); all of them Nycteribiidae, with the exception of the streblid genus *Trichobius*. Both host fly families displayed a similar distribution of microparasite taxa (Figure 4).

The most commonly reported microparasites in bat flies are bacteria (*n* = 149), followed by fungi (*n* = 118), blood parasites (*n* = 15), viruses (*n* = 6), and arthropods (*n* = 1) (Table 1). Within bacteria, the three most frequently detected microparasites are *Bartonella* sp. (Alphaproteobacteria: Bartonellaceae) (*n* = 91, 61%), *Arsenophonus* sp. (Gammaproteobacteria: Enterobacteriaceae) (*n* = 30, 20.1%) and *Wolbachia* sp. (Alphaproteobacteria: Anaplasmataceae) (*n* = 8, 5.4%). All observed fungi are Laboulbeniaceae (Ascomycota: Laboulbeniales) and belong to three genera, *Arthrorhynchus* (*n* = 80, 67.8%), *Gloeandromyces* (*n* = 16, 13.6%), and *Nycteromyces* (*n* = 5, 4.2%), as well as 17 (14.4%) unidentified or undescribed observations. *Polychromophilus* species (Haemosporida: Plasmodiidae) represent 93.3% (*n* = 14) of blood parasite observations in bat flies. Virus and parasitoid arthropod represent a much smaller proportion of all microparasitic observations in bat flies, with only six and one published record, respectively.

**Table 1:** Microparasite groups found in bat flies and their associated bat families.

**Bat host family**	***N* of observation**	***N* of bat fly species with microparasites**	**Microparasites detected from flies**	***N* of observation**	**Location**	**References**
Emballonuridae	1	1	Blood parasites	1	Gabon	(63)
Hipposideridae	7	6	Bacteria	2	Gabon, Malaysia	(30, 44)
			Fungi	3	Sri Lanka, Zambia	(64–67)
			Blood parasites	2	Gabon	(63)
Miniopteridae	57	14	Bacteria	21	Hungary, Japan, Madagascar, Romania	(26, 46, 68)
			Fungi	29	Australia, Bulgaria, Croatia, France, Hungary, India, Kenya, Portugal, Romania, Slovakia, Spain, Sri Lanka, Switzerland, Taiwan	(64, 66, 69–76)
			Blood parasites	7	Gabon, Madagascar	(63, 77, 78)
Mormoopidae	3	2	Bacteria	1	Mexico	(44)
			Fungi	2	Costa Rica, Panama	(79)
Noctilionidae	2	2	Bacteria	2	Dominican Republic, Panama	(28, 44)
Phyllostomidae	48	18	Bacteria	18	Brazil, Costa Rica, Dominican Republic, French Guyana, Mexico, Panama, Peru	(27, 45, 80, 81)
			Fungi	28	Brazil, Costa Rica, Grenada, Panama, Venezuela	(64, 74, 79, 82–86)
			Virus	2	Mexico	(87)
Pteropodidae	35	17	Arthropod	1	São Tomé Island	(88)
			Bacteria	23	China, Gabon, Ghana, Kenya, Madagascar, Malaysia, Philippines, Union of the Comoros	(12, 28, 30, 44, 53, 68, 89)
			Fungi	6	Egypt, Gabon, Israel, Malaysia, New Guinea, Sierra Leone	(66, 71, 90, 91)
			Blood parasites	1	Gabon	(63)
			Virus	4	China, South Africa, Uganda	(92–96)
Rhinolophidae	21	7	Bacteria	8	China, Hungary, Laos, Philippines, Romania	(28, 44, 46)
			Fungi	13	Croatia, France, Hungary, Italy, Kenya, Romania, Serbia, Sri Lanka	(64, 66, 67, 97)
Vespertilionidae	58	19	Bacteria	34	Costa Rica, Hungary, Madagascar, Malaysia, Peru, Romania, Slovenia, United States	(28, 30, 44, 46, 47, 50, 68, 81, 98)
			Fungi	21	Austria, Brazil, Czech Republic, England, France, India, Italy, Poland, Portugal, Romania, Spain, Tunisia	(64, 66, 71, 75, 76, 83, 99–101)
			Blood parasites	3	England/Scotland, Switzerland	(102, 103)

*The number of microparasite and host species associations (both bat species and bat fly species) are given, as well as the country of observation. See references and additional details in Supplementary Data Sheet 1*.

### Global Geographical Distribution of Bat Fly—Microparasite Associations

**Figure 5:**
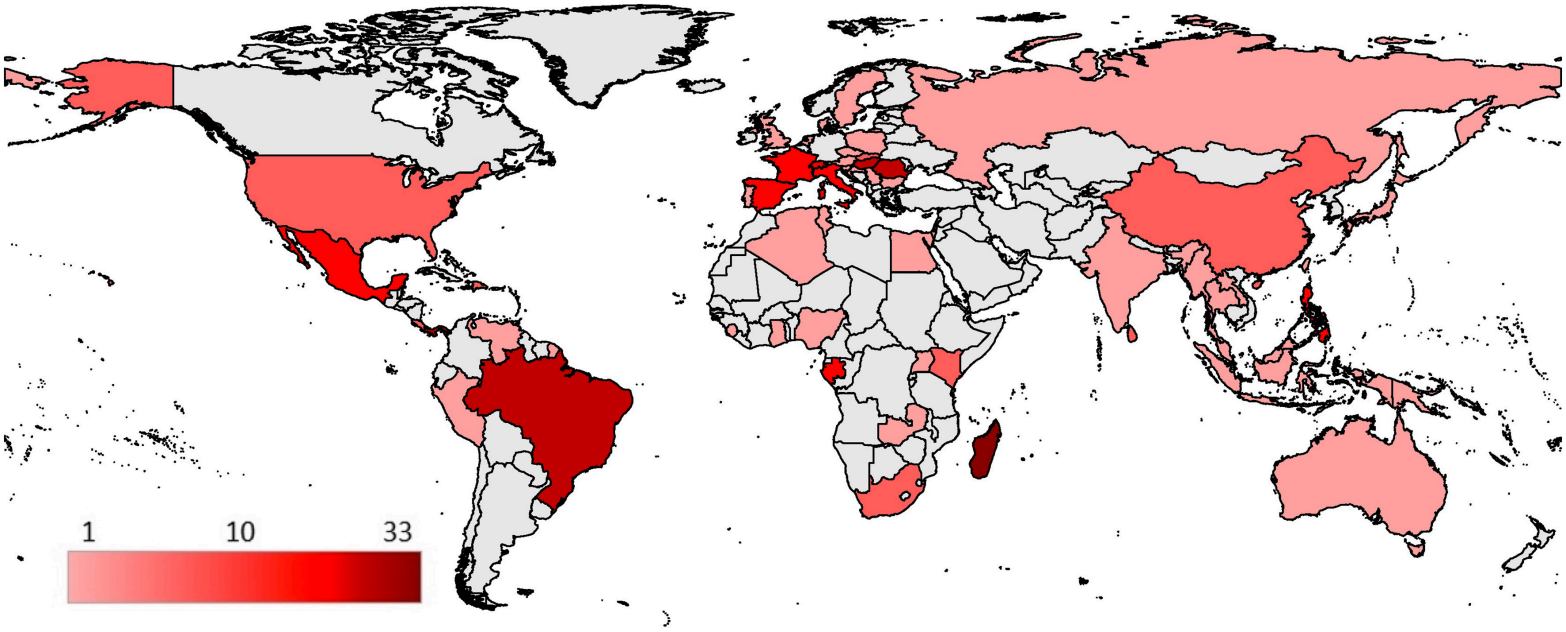
Geographical distribution of reported bat fly—microparasite species associations. Countries are colored according to the number of different described species associations.

Bat fly -microparasite associations originated from 61 countries (Figure 5) with a total of 269 reports (excluding those with unspecified or unknown geographical locations). Associations reported from countries were most commonly from Europe (*n* = 89, 33%), North America (*n* = 69, 25.7%), and Africa (*n* = 61, 22.7%). Observations in Asia (*n* = 33, 12.3%), South America (*n* = 21, 7.8%), and Oceania (*n* = 5, 1.9%) were represented less frequently. The highest number of microparasite—bat fly species associations are reported from Madagascar (*n* = 33).

### Sampling Effort on Microparasite Diversity in Bat Flies

**Figure 6:**
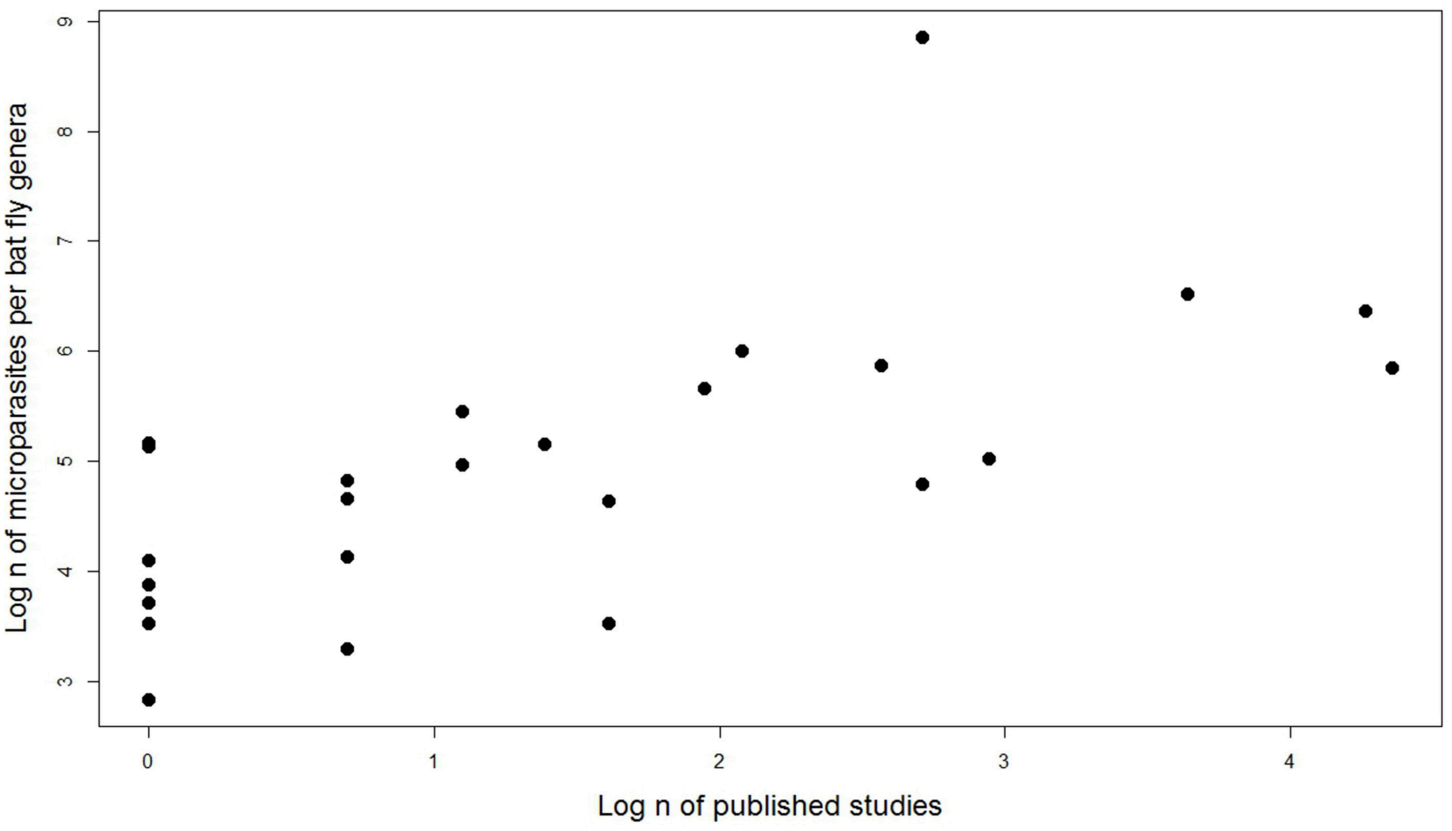
Effect of sampling effort on the number of microparasite associations in different bat fly genera.

We tested the number of published studies by bat fly genera and number of microparasite associations reported (including same species associations but different bat hosts and countries). Spearman rank correlation showed that sampling effort strongly predicts the number of detected microparasites in different bat fly genera (*n* = 27, *r* = 0.68, *p* = 0.0001; Figure 6).

## Discussion

### Microparasite Diversity in Bat Flies

Based on literature data, we have identified five main groups of microparasites in parasitic bat flies. Bacteria are the most frequently observed group in both Nycteribiidae and Streblidae and within bacteria, *Bartonella* is the most prevalent microorganism. Some species of *Bartonella* are blood-borne parasites, transmitted by blood-sucking arthropods (104) found in a wide range of mammalian groups and several arthropod ectoparasites (14). For example, *Bartonella quintana*, a louse-borne parasite, was responsible for trench fever, which affected over one million soldiers during World War 1 (105). The presence of identical *Bartonella* genotypes in bats and bat flies suggests that bat flies may serve as vectors (44, 53, 80, 81). Host specific bat flies show higher prevalence but lower diversity of *Bartonella* infection than polyxenous species (46). However, the generally high prevalence and diversity of *Bartonella* suggests their long co-evolutionary history with bats.

The second most frequently observed microparasites in bat flies are fungi. All species recognized here belong to the order Laboulbeniales. Three genera of Laboulbeniales are known to parasite bat flies, *Arthrorhynchus* spp. (the most frequently reported genus), *Gloeandromyces* spp., and *Nycteromyces* spp. The distribution, specificity and diversity of these microparasites have recently been uncovered. Locally (e.g., in Europe) these species show some degree of high specificity (with occasional “accidental” transfers) (64, 69), although at a larger geographical scale, they do not show strict specificity to host species or genera (65).

While blood parasites are frequently found in bats (77, 106–108), observations in bat flies are much less common. *Polychromophilus* species are vectored by nycteribiids (102), and one haemosporidian report is known from a single fossil streblid specimen but observations from extant streblids are still missing (109). Other blood parasites, such as *Trypanosoma* is transmitted to bats by hemipterans including *Cimex* species (42). *Trypanosoma cruzi cruzi*, the causative agent of Chagas disease in humans and other mammal species, is transmitted by triatomine bugs (110). Bat flies have not yet been reported as vectors of *Trypanosoma* species. Nevertheless, *Glossina* tsetse flies (members of the Hippoboscoidea superfamily along with bat flies) are known to transmit *T. brucei*. Therefore, it remains possible that bat flies transmit other blood parasites besides *Polychromophilus* (e.g., trypanosomatids). Additionally, nycteribiids may serve as vectors in the transmission of other protozoans, such as *Nycteria* spp. (Haemosporida: Plasmodiidae), infecting Afrotropical insectivorous bats; but their vectorial potential has not yet been clarified (107). More work is needed to address these questions.

Most of the reports on viruses in bat flies are relatively recent (87, 92–96). As such, it is possible that the number of isolated viruses in bat ectoparasites might thus rise in the future with improvement in diagnostic methods.

There is only one report of a parasitoid wasp using nycteribiids as host (88). Parasitoid wasps are extremely diverse groups with about 100,000 described species. However, host species information is missing for many species. We expected that other parasitoids use bat flies as hosts during their development, but data collection is challenging due to the ecology of these flies. Furthermore, it has been observed that mite species can have phoretic relationships with bat flies (111–113), but their effect on bat flies is not clear. Nonetheless, some phoretic mites which were previously assumed to have no effect on their invertebrate hosts, have now been shown to negatively affect their fecundity and/or survival rate (114, 115).

Studies have previously suggested that microfilaria might be transmitted by hippoboscid louse flies to their vertebrate hosts, such as dogs (116). Filarial nematode DNA has also been observed in streblid bat flies and bat mites (117). It is not clear if these microfilaria are transmitted by bat flies or if the detected microfilaria DNA was only present in the last blood meal (117).

Microparasite diversity is similar between nycteribiids and streblids flies, although nycteribiids have 2.5 times more reported cases of microparasites. The reason behind this is more likely due to biased sampling efforts in different geographical regions. For example, in Europe where most of the studies were performed, 16 species of nycteribiids are present, whereas only one streblid species have been recorded.

### Geographical Distribution

All major groups of microparasites have been reported widely, though our knowledge of the diversity and distribution of many groups remains scarce. Bacteria such as *Bartonella* show a high molecular and geographic diversity in bats and bat flies, at global and regional scales (44, 46, 118). Six major bat associated *Bartonella* clades have been reported so far from bats and bat flies (118). Clade I, II, IV, and V are represented in both Old and New World areas while clade III seems to be restricted to the Old World (Africa, Asia, and Europe) and clade VI to some parts of the New World (Central America) (118).

Fungal microparasites (Laboulbeniales) show a rather divided Eastern (*Arthrorhynchus* spp.) and Western Hemisphere (*Nycteromyces* spp. *Gloeandromyces* spp.) distribution and diversity (65). Similar patterns have been demonstrated regarding nycteribiids (Eastern) and streblids (Western) (17). These diversity and distribution patterns suggest a long evolutionary history between bat flies and these fungal microparasites.

It is important to highlight that these distribution patterns might be strongly influenced by biased sampling efforts rather than actual geographical patterns. Therefore, the distribution map helps to recognize well studied areas on a global scale, however it does not necessarily reflects actual distributional patterns of these microparasites detected in bat flies. It is our hope that it will be useful for further studies.

### Effects of Bat Host Ecology on Microparasites

Previous work showed that viral richness in bats correlates with IUCN threat status, with near-threatened and vulnerable hosts having higher viral richness. In addition, population genetic structure positively correlates with viral richness (119). Host longevity, reproductive strategy and distribution pattern may also play an important role in viral richness (9, 54, 120).

In general, the bat host family does not affect the distribution of microparasites in their bat flies. The bent-winged bats, family Miniopteridae, have the highest observed ratio of bat species infected by bat flies parasitized by microparasites. Miniopteridae are insectivorous, cave-dwelling species occurring in dense and multi-species colonies. From a disease ecology and parasitology point of view, it is a unique family hosting many highly specific ecto- and endoparasites such as mites, bat flies and malarial parasites (21, 121, 122). It is still unclear whether the ecology and/or the immune system of Miniopteridae species is responsible for such a high parasite diversity compared to other bat families. Moreover, Miniopteridae is considered as underrepresented in viral research so more parasites and pathogens likely remain undiscovered in these species (123).

Bacteria and fungi are the most abundant group of microparasites in all bat flies from different host families. The occurrence of *Bartonella* infection in bats is associated with host diet; hematophagous and carnivorous species are more frequently infected than species with other diets (124). Hematophagous and carnivorous bat species also show higher white-blood cell count, suggesting a higher risk of pathogen exposure, probably due to the fact that these bat species are more exposed to vertebrate specific pathogens (125). Therefore, we might expect a higher microparasite occurrence in bat flies collected from bat species that feed on vertebrates or blood. Nevertheless, there are only a few studies that have focused on microparasites in parasitic bat flies collected from these host species (44, 80, 87).

Viruses are only known from bat flies infecting the New World leaf-nosed bats Phyllostomidae and the Old World fruit bats Pteropodidae, but observations are still scarce. These observed viruses represent distant groups, such as Dengue virus (family *Flaviviridae*) isolated from the bat flies of the common vampire bat, *Desmodus rotundus* (87); Kaeng Khoi virus (*Peribunyaviridae*), Kanyawara virus (*Rhabdoviridae*), Mahlapitsi virus (*Reoviridae*), and Wolkberg virus (*Bunyaviridae*), isolated from *Myonycteris* and *Rousettus* species (92–96).

There are great ecological differences between bat families. Bat host ecology and physiology, such as roosting habits, body size, and sex can affect bat fly burden and species richness (126–129). More studies are again needed to clarify how host traits affect the distribution of microparasite communities of bat flies.

### Potential Physiological Effects on Flies and Bats

We still know little about the physiological effects of microparasites on bat flies and on their bat host. Viruses such as *Lyssavirus* spp. are known to cause mortality in bats (130, 131). The bacterial parasite *Borellia* sp. (from the relapsing fever group) has been documented causing fatal borreliosis in a single bat individual (*Pipistrellus* sp.) (132). The haemosporidian parasite *Polychromophilus murinus* has a well-documented impact on both bat and bat fly life-history traits (103, 106). In the Daubenton's bats (*Myotis daubentonii*), it has a strong negative effect on the body condition of subadults (106). Additionally, it negatively affects the life span of infected bat flies (103).

The relationship between bat flies and some bacterial species such as *Wolbachia* and *Arsenophonus* has not yet been clarified. It is suspected that they are either parasitic and/or symbiotic of bat flies. In some cases, *Wolbachia* is considered as a nutritional mutualist, due to its ability to produce vitamin B in certain hematophagous arthropod species, such as *Cimex* spp. (133). *Arsenophonus* is a highly diverse group of bacteria found mainly in insects, including bat flies (134–138). *Arsenophonus* species have been suggested to be primary or secondary symbionts in other taxa (134, 138, 139). Here, we categorize *Arsenophonus* and *Wolbachia* as microparasitic organisms in bat flies, since it is unclear how they affect their hosts (35). Furthermore, *Wolbachia* DNA has been also detected in mammalian blood due to the presence of infected nematodes in host blood (140). It has been observed once in an avian blood system, with the strain being more closely related to the arthropod-associated *Wolbachia* group (141), and likely having no direct effect on their vertebrate hosts.

The presence of the fungal parasite Laboulbeniales has an effect on bat fly mortality in some species (Szentiványi et al., Unpublished), as an arthropod specialized microparasite. Nevertheless, it is unclear if it has any direct or indirect effect on the bat host.

Additionally, and as mentioned above, the potential effect of phoretic mite infestation on bat flies has never been tested. Therefore, it remains possible that these mites have direct or indirect negative effects on host behavior, survival rate, and/or fecundity.

### Perspectives for Additional Research, Sampling Effort

Our knowledge of the microparasites of bat flies is strongly biased by sampling effort, which may also strongly reflect the currently known geographical distribution patterns of these parasites. We suggest to balance these biases by increasing sampling effort in less prospected countries as well as areas where human exposure to pathogen transmission is more likely to occur, due to cultural or touristic reasons (e.g., visiting caves) (15, 142). Additionally, we have little knowledge on the microparasites of other bat ectoparasitic groups, such as fleas, bugs, and mites. Future studies should focus on how microparasite and pathogen communities interact on the intra- and interspecific levels. For example, *Wolbachia* infection is known to inhibit malarial infection in mosquitos (143). Additionally, it is important to understand how bat host traits such as sex, geographical distribution and/or host group size [which are known to shape the distribution of bat fly populations (17, 128, 129)] may affect the occurrence of microparasitic communities in these ectoparasites. Lastly, experimental studies are needed to understand the relationship between bat hosts and ectoparasites, including the transmission and the distribution of microparasites.

## Author Contributions

PC and OG initiated the study. TS performed data collection and wrote the first draft of the manuscript. All authors read and approved the final manuscript.

### Conflict of Interest Statement

The authors declare that the research was conducted in the absence of any commercial or financial relationships that could be construed as a potential conflict of interest.
